# Effects of Microalgae on Metabolic Syndrome

**DOI:** 10.3390/antiox12020449

**Published:** 2023-02-10

**Authors:** Kartthigeen Tamel Selvan, Jo Aan Goon, Suzana Makpol, Jen Kit Tan

**Affiliations:** Department of Biochemistry, Faculty of Medicine, Universiti Kebangsaan Malaysia (UKM), Jalan Ya’acob Latiff, Bandar Tun Razak, Cheras, Kuala Lumpur 56000, Malaysia

**Keywords:** microalgae, metabolic syndrome, obesity, hypertension, hypertriglyceridemia, high-density lipoprotein cholesterol, hyperglycemia, type 2 diabetes mellitus, cardiovascular disease

## Abstract

Metabolic syndrome (MetS) is a cluster of metabolic disturbances, including abdominal obesity, hypertension, hypertriglyceridemia, reduced high-density lipoprotein cholesterol (HDL-C) and hyperglycemia. Adopting a healthier lifestyle and multiple drug-based therapies are current ways to manage MetS, but they have limited efficacy, albeit the prevalence of MetS is rising. Microalgae is a part of the human diet and has also been consumed as a health supplement to improve insulin sensitivity, inflammation, and several components of MetS. These therapeutic effects of microalgae are attributed to the bioactive compounds present in them that exhibit antioxidant, anti-inflammatory, anti-obesity, antihypertensive, hepatoprotective and immunomodulatory effects. Therefore, studies investigating the potential of microalgae in alleviating MetS are becoming more popular, but a review on this topic remains scarce. In this review, we discuss the effects of microalgae, specifically on MetS, by reviewing the evidence from scientific literature covering in vitro and in vivo studies. In addition, we also discuss the underlying mechanisms that modulate the effects of microalgae on MetS, and the limitations and future perspectives of developing microalgae as a health supplement for MetS. Microalgae supplementation is becoming a viable approach in alleviating metabolic disturbances and as a unique addition to the management of MetS.

## 1. Introduction

Metabolic syndrome (MetS) is a cluster of metabolic disturbances including abdominal obesity (elevated waist circumference), hypertension, hypertriglyceridemia, reduced high-density lipoprotein cholesterol (HDL-C) and hyperglycemia, which are some of the major risk factors for developing cardiovascular disease (CVD) and type 2 diabetes mellitus (T2DM) [1]. For MetS to be clinically diagnosed, one must have at least three of those metabolic disturbances [1,2,3,4,5]. The occurrence of non-alcoholic fatty liver disease (NAFLD), which is defined by an excessive hepatic accumulation of triglycerides and cholesterol gallstone disease, are two main manifestations of MetS in the liver [1,6]. Moreover, insulin resistance (IR) is one of the crucial factors involved in the etiology of MetS and its comorbidities due to the important role of insulin in regulating energy, glucose, and lipid metabolism [1,7,8]. Therefore, IR is also involved in the pathogenesis of NAFLD, T2DM, obesity and CVD [9]. These conditions are connected by low-grade inflammation, which is a key pathophysiological process in the pathogenesis and growth of disturbances associated with MetS [10]. In that context, the release of pro-inflammatory cytokines and adipokines by immune cells and adipose tissues mediate the state of inflammation in the body leading to IR, exacerbation of NAFLD and development of CVD [9,10].

The prevalence rate of MetS differs across regions, which also depends on the diagnostic criteria used, age, gender, race, and ethnicity. In the US, 34% of adults are diagnosed with MetS. This percentage is growing strikingly along with the prevalence in other regions such as Europe and Asia [1,11]. The morbidity and mortality of MetS have significantly risen in recent years, and it now accounts for 17.3 million deaths per year globally. This number is projected to increase to approximately 23.6 million by 2030. The rise in global MetS cases is concurrent with the increasing global rates of obesity that have almost tripled in the past 50 years according to the World Health Organization (WHO) [11]. Obesity is also increasing in children and adolescents; thus, leading to an increase in the prevalence of MetS among these youngsters [1]. The prevalence of T2DM and CVD is also rising and MetS could further increase their high prevalence and mortality rates [11]. The worldwide increase in MetS is driven by a sedentary lifestyle and excessive calorie consumption. The management is focused on adopting a healthier lifestyle such as a healthy diet and increasing physical activity but adherence to these management plans is low and the long-term benefits remain elusive [12]. To date, there have been no established pharmaceutical treatment options for treating MetS as a whole, so current strategies are only able to treat individual components of MetS using multiple drug-based therapy regimes. However, such regimes can be complicated, so it is challenging to comply with, and the efficacy of the drugs is limited due to their adverse side effects and drug-drug interactions [1,13,14]. Therefore, the lack of treatment options and increasing MetS prevalence describe the need to explore novel sources of bioactive substances with the potential for medicinal and/or preventative effects.

Microalgae are unicellular and photosynthetic microscopic algae that live in fresh or saltwater which comprise eukaryotic microalgae and prokaryotic cyanobacteria [15,16]. From the human consumption perspective, microalgae are cultivated commercially for application in the food and cosmetic industries. Microalgae biomass and their extracts, such as pigments, carotenoids, fatty acids and proteins, have been incorporated as additives or ingredients in food products such as biscuits, bread, cookies, snacks, milk, yogurt, and pasta [17,18,19,20]. Microalgae are emerging sources of bioactive components such as polysaccharides (especially β-1,3-glucan), carotenoids, pigments and water extracts in cosmetic products namely lotions, creams, shampoos, and soaps that are marketed with anti-aging, UV protective and moisturizing properties [20,21,22,23]. Eukaryotic microalgae such as *Chlorella* sp., *Dunaliella salina*, *Haematococcus pluvalis*, *Crypthecodinium cohnii* and *Shizochytrium*, and prokaryotic cyanobacteria such as *Arthrospira plantesis* and *Aphanizomenon flos-aquae* are the most popular microalgae species used for human consumption but many other species are yet to be explored [20,24,25]. 

In this era, poor diet choices that lack antioxidants, polyunsaturated fatty acids (PUFAs), fibers, vitamins, and minerals together with unhealthy habits, lack of physical exercise and poor sleep quality have greatly contributed to the increase of many non-communicable diseases [8,26,27]. To address this issue, microalgae and their bioactive compounds have been researched for their potential as health supplements [20], one of which is microalgae fatty acids. Although all microalgae species contain C16, C18 and monounsaturated fatty acids (MUFAs), PUFAs such as eicosapentaenoic acid (EPA) and docosahexaenoic acid (DHA), which are mainly present in marine species and some freshwater species, are of most interest. In addition to fatty acids, some species contain bioactive compounds such as pigments, vitamins, mycosporine-like amino acids (MAAs), scytonemins, pterins, phenolic compounds, carotene and xanthophylls. However, the composition of these bioactive compounds including fatty acids, protein and polysaccharides vary among different microalgae species [28]. These compounds in microalgae have exhibited antioxidant, anti-inflammatory, anti-obesity, anti-viral, anticancer, antihypertensive, hepatoprotective and immuno-modulatory activity [20,28,29]. Moreover, several studies have found that microalgae supplementation in MetS-induced models improved insulin sensitivity, inflammation, and several components of MetS such as obesity, hypertension and dyslipidemia that reveal its potential in alleviating metabolic disturbances associated with MetS [24]. These findings have encouraged the commercialization of certain microalgae species as health supplements in capsules, tablets, powders, or liquids. Among various species, *Spirulina* sp. and *Chlorella* sp. have gained the highest traction in the global microalgae market for health supplements [20]. *A. flos-aquae*, *Tetraselmis chuii*, *Odontella aurita*, *Phaeodactylum tricornutum*, β-carotene from *Dunaliella*, DHA and EPA from *C. cohnii* and *Schizochytrium* sp., and astaxanthin from *H. pluvialis* are also commercially available as health supplements [20,28,29,30,31]. Most studies showing the therapeutic effect of microalgae, such as its fatty acids on MetS, have used microalgae extracts, powders or oils that were prepared in labs for research purposes. However, microalgae oils rich in DHA are commercially available and have been used for research to determine their therapeutic effects [32,33,34,35]. Although microalgae seem like a promising option for alleviating diseases, there are no microalgae-based products approved by FDA as therapy in disease management. The therapeutic potentials of some microalgae and their bioactive compounds as drug candidates remain up to clinical trials and require further investigations [36,37]. 

Nevertheless, there has been an increasing number of studies investigating the effect of microalgae as a health supplement for MetS but a review on this topic remains scarce. Therefore, this review will gather the evidence from scientific literature covering in vitro, in vivo, and possibly human studies that report the effects of microalgae on MetS. We will also incorporate the mechanisms that modulate the effects of microalgae on MetS. Lastly, the limitations and future perspectives of developing microalgae as a health supplement for Mets will be covered in this review too.

## 2. Effect of Microalgae on MetS

### 2.1. In Vitro Study

A cell model representing MetS is yet to be reported in the literature, probably because some MetS criteria are improbable to be measured in a cell-based study. Interestingly, a study has assessed the microalgae-derived products in several cell models that closely recapitulate some MetS criteria in a well-designed study that compared with the positive control drugs (Table 1).

#### Effects of Oxohexadecenoic Acids Derived from *Chaetoceros karianus* on MetS

The Peroxisome proliferator-activated receptors (PPARs) such as PPARγ and PPARα, are types of nuclear receptors found in the human liver and adipose tissue, respectively. These are ligand-activated receptors that heterodimerize with retinoid X receptors (RXRs) to regulate gene expression by acting as transcriptional factors [38]. Activated PPARγ modulates the growth and differentiation of adipocytes through the upregulation of lipogenesis and lipid storage genes, and functions to regulate insulin sensitivity by promoting the synthesis of adiponectin and leptin, and improvement of hyperglycemia [39,40,41,42]. In contrast, activated PPARα is involved in the uptake, activation, and oxidation of fatty acids in the liver [41]. Impairment in these PPARs is related to obesity, IR and diabetes mellitus, which are some of the conditions associated with MetS [40]. PPAR agonists such as thiazolidinedione (targets PPARγ) and fibrates (targets PPARα) have been proven to improve insulin sensitivity and hyperlipidemia, respectively [39,40,41,42].

Long-chain fatty acids such as oxo-fatty acids (oFAs) are involved in different physiological processes that appear to be connected to the pathophysiological state caused by obesity such as IR, diabetes, and cardiovascular diseases [43,44,45]. A study has found that oFAs produced by gut bacteria exhibited PPARγ ligand activity which may be used to modulate the host’s energy metabolism [45]. In line with this, (7E)-9-oxohexadec-7-enoic acid [(7E)-9-OHE] and (10E)-9-oxohexadec-10-enoic acid [(10E)-9-OHE], which are two novel oxo-fatty acids identified from the microalgae *C. karianus*, have been reported to exhibit considerable PPARα/γ agonist activity [41]. Another study found that OFAs produced by gut bacteria have previously been shown to promote the secretion of cholecystokinin (CCK) to improve postprandial glycemia, lipidemia, and appetite but its PPAR agonist activity was not assessed [46].

Sæther et al. investigated the potential of oFAs, [(7E)-9-OHE] and [(10E)-9-OHE] from *C. karianus* in activating PPAR in hepatocytes and adipocytes [38]. Preliminarily, the dose-dependent effects of both oFAs were studied in COS1 cells (a fibroblast-like cell line derived from African green monkey kidney) treated with (7E)-9-OHE, (10E)-9-OHE, pirinixic acid, rosiglitazone, or palmitic acid for 18 h. The results show that both oFAs produce considerable dose-dependent PPAR agonist activity. In that context, both oFAs demonstrated similar potency as pirinxic acid, which is a positive control, in activating the PPARα receptor. As for PPARγ activation, the oFAs had lower potency and efficacy compared to rosiglitazone which is another positive control but the PPRAγ activation was still higher compared to palmitic acid being the negative control. To determine the endogenous PPAR target genes activated by the oFAs in the liver, Huh-7 was treated with (7E)-9-OHE or (10E)-9-OHE. The results showed that the oFAs treatment was able to activate fatty acid catabolism in hepatocytes. In that context, the oFAs upregulated the expressions of CPT1A and ANGPTL4 due to the activation of the PPARα receptor. Moreover, ACSL3 and PLIN1 genes were significantly upregulated by treating the cells with (7E)-9-OHE and (10E)-9-OHE, respectively. CPT1A gene functions to synthesize the CPT1 enzyme, which is the rate-limiting enzyme for fatty acid β-oxidation, and the ACSL3 gene encodes for acyl-CoA synthetases that convert FAs into fatty acyl-CoA esters which participate in β-oxidation [47,48]. Besides that, the ANGPTL4 gene functions to modulate TG and energy homeostasis according to body state [48,49,50]. ANGPTL4 expression has been shown to improve glucose tolerance and hyperglycemia in diabetic mice which suggests that it can improve glucose metabolism [51]. Lastly, PLIN1 is involved in modulating lipolysis depending on energy requirement, so these genes also regulate lipid metabolism in the liver [52]. The endogenous PPAR target genes activation by the oFAs was also determined in SGBS adipocytes. The SGBS cells were originally pre-adipocytes that were differentiated in an adipogenic medium for 8 days. At day 8, the SGBS cells were stimulated with (7E)-9-OHE, (10E)-9-OHE or rosiglitazone medium for 24 h. The results show that both oFAs induce fatty acid catabolism in adipocytes by upregulating CPT1A expression. In adipose tissue, CPT1A improves fatty acid-induced IR and reduces TG accumulation [47]. Moreover, in SGBS cells treated with (10E)-9-OHE, ANGPTL4 expression was upregulated. Apart from its effect on TG metabolism, ANGPTL4 also functions as an adipokine in inducing adipocyte differentiation and adipose tissue expansion [49]. Overall, the findings suggest that oFAs play a role in regulating adipocyte metabolism but are not as efficacious as the drug, rosiglitazone.

Next, the study compared the adipogenic potentials of the oFAs with rosiglitazone. Here, the SGBS cells were differentiated in a medium containing (7E)-9-OHE, (10E)-9-OHE or rosiglitazone for 12 days in which the medium was renewed every 4 days. The results show that both oFAs increased the number of adipocyte-like cells and total volume of lipid droplets upon Oil-Red-O staining but not as profound as rosiglitazone-treated cells. The author stated that the RNA analysis suggests that the oFAs were able to promote the regulation of fatty acid metabolism, transport, storage, adipokine signaling and browning. In that context, the oFAs upregulated PPARG, CEBPA, CEBPB, PLIN1, FABP4, CD36 and SCD1 gene expressions. CEBPB promotes the expression of PPARG and CEBPA, being the key event in adipocyte differentiation [53]. Meanwhile, the FABP4 gene functions to maintain adipocyte homeostasis [54], while the CD36 gene may play a role in modulating adipocyte hyperplasia and hypertrophy [55]. SCD1 gene produces the rate-limiting enzyme in the synthesis of MUFAs involved in lipogenesis [56]. So, the upregulation of these genes favors adipogenesis. However, these gene expressions were 5 to 10-fold higher with rosiglitazone. The author suggested that with oFAs treatment, only temporary upregulation of crucial adipogenic genes such as PPARG and C/EBPa genes was possible but a more stable expression is exhibited when treated with rosiglitazone. Furthermore, the oFAs upregulate UCP1 gene expression, although not as profoundly as rosiglitazone. The UCP1 gene is crucial in the event of adipose tissue (AT) browning [53]. Adipose tissue can be divided into white and brown. White adipose tissues (WAT) function to store excess energy as TG and release adipokines that are involved in the regulation of energy metabolism. Moreover, brown adipose tissue (BAT) functions as a thermogenic organ and protects against obesity and certain metabolic disturbances. Therefore, adipose tissue dysfunction and/or imbalance in their composition can lead to metabolic derangements [57,58]. Hence, these oFAs have the potential to induce adipose tissue browning that is associated with improvement in dyslipidemia and obesity.

To further reveal the difference between the oFAs and rosiglitazone in stimulating adipocyte differentiation, adipocyte transcriptomic analyses were included in this work. As a result, it was shown that the adipogenic effects of the oFAs were considerable but still lower compared to rosiglitazone. This may not appear ideal since fatty acid uptake and adipogenesis are important effects exerted by PPARγ agonists in the attempt to alleviate dyslipidemia. However, considering the side effects of thiazolidinediones (rosiglitazone) such as weight gain and edema [40], oFAs are still potential substitutes. Furthermore, the oFAs were able to upregulate the expressions of leptin and the insulin-sensitizing adipokine adiponectin (ADIPOQ) genes. Moreover, PPARγ activation by the oFAs is associated with the suppression of pro-inflammatory cytokines gene expressions such as IL-6, TNFa, CXCL1, CXCL5 and IL-1B. Additionally, the expression of IRS1 and SLC2A4 genes was increased. Increased expression of IL-1B is linked to the downregulation of IRS1 and SLC2A4 genes which leads to reduced adipose insulin sensitivity. Therefore, these oFAs are termed as semi-potent dual PPARα/γ agonists and activate anti-diabetic gene programs via suppressing pro-inflammatory cytokines and increasing insulin-sensitive adipokines. As such, dual PPARα/γ agonists activity of the oFAs has the potential to alleviate hyperglycemia and dyslipidemia which are crucial components of MetS.

### 2.2. In Vivo Studies

Many studies have reported the effects of microalgae on the individual components of MetS using obese, diabetic and hypertension animal models. However, these models might not be the best to represent MetS because the status of other components was not evaluated or might not be affected by the component-specific models. Hence, the actual therapeutic potential of microalgae on MetS is difficult to appreciate. Therefore, this review will only include those studies that have established the MetS models by measuring most if not all the parameters that are sufficient to define the presence of MetS and discuss the effects of microalgae on these components (Table 2).

#### 2.2.1. *Tetraselmis chuii*

Gil-Cardoso et al. investigated the potential therapeutic effects of *Tetraselmis chuii* (eukaryotic microalgae) using a 100% lyophilized *T. chuii* powder, marketed under the name “TetraSOD*^®^*” in a cafeteria (CAF) diet-induced MetS rat model [59]. Seven week old Sprague Dawley male rats were assigned to five groups: standard diet-control (STD-C), cafeteria diet-control (CAF-C), CAF diet for the first 8 weeks and supplemented with 0.17, 1.7 or 17 mg/kg BW/day of *T. chuii* powder for the next 8 weeks (CAF + 0.17, CAF + 1.7 or CAF + 17). CAF diet consists of foods that are often considered to be delicious but unhealthy such as food high in salt, fat, low in fiber and energy-dense foods. In comparison with a typical high-sugar, high-fat diet, the CAF diet has a better variety of sensory properties that induce hyperphagia, so it can better recapitulate unhealthy eating patterns. Thus, the CAF diet is better at inducing MetS and diabetic complications in addition to inducing obesity compared to high-sugar, high-fat diets [65,66]. However, the CAF diet might lack adequate protein and essential micronutrients which can result in nutritional deficiencies. In this experiment, the CAF diet contained higher total fat, saturated fat and sugar compared to the standard diet (STD). It was reported that BW, adiposity index, TG, and IR measured by Homeostatic Model Assessment for IR (HOMA-IR) were increased by the CAF diet but these parameters were not affected by *T. chuii* supplementation. However, a decrease in the plasma glucose level and low-density lipoprotein-cholesterol (LDL-C)/very low-density lipoprotein-cholesterol (VLDL-C) were observed in CAF + 17 and CAF + 0.17, respectively, which is a beneficial effect when it is taken into account that a high plasma glucose level is a risk factor for developing MetS. Nevertheless, the HDL-C levels were not affected by the supplementation.

The author stated that the therapeutic effects of *T. chuii* on the CAF-induced MetS rats were due to its antioxidant, anti-inflammatory and immune system-modulating properties. In that context, the plasma levels of antioxidant and inflammation markers such as the plasma-oxidized low-density lipoprotein (oxLDL) level decreased in CAF + 17 compared to STD-C and CAF-C. A decreasing trend of the oxLDL levels was observed from the lowest to the highest concentrations of *T. chuii* treatment but these changes are not significant from one another. Furthermore, plasma nitric oxide metabolites (NOx) increased in CAF + 0.17 compared to STD-C and CAF-C. The author stated that NOx regulates endothelial function and is involved in inflammation and inhibiting atherosclerosis [67]. Meanwhile, plasma anti-inflammatory cytokine interleukin-10 (IL-10) levels were increased in CAF + 17 compared to CAF-C. IL-10 suppresses the expression of genes involved in the production of pro-inflammatory cytokines such as interleukin-1 beta (IL-1β) and tumor necrosis factor α (TNF-α) [68,69]. The liver counteracts damages due to oxidative stress by the elevation of antioxidant enzymes such as glutathione peroxidase (GPx) and glutathione (GSH) [70]. In this study, GPx activity is shown to have increased in the livers of CAF + 0.17 rats. The CAF diet indeed decreased the GSH levels in CAF-C, but the levels increased in the livers of CAF + 17 rats. Hence, *T. chuii* treatment promotes the production of antioxidant enzymes to reduce oxidative stress associated with MetS.

To elucidate the underlying mechanism of these effects, the author performed gene expression studies on the genes involved in GSH metabolism. The synthesis of GSH involves the actions of glutathione reductase (GR), glutathione synthetase (GSH-S) and glutamate–cysteine ligase (GCL) enzymes. In the liver, *T. chuii* supplementation caused the upregulation of GR and GSH-S genes. GCLm, one of the subunits of the GCL enzyme has shown an increase in its gene expression in the two highest dosages as compared to CAF-C rat livers. The GPx enzyme which is encoded by the GPX1 gene forms the liver antioxidant defense together with GSH and superoxide dismutase (SOD) enzymes. The results revealed that GPx1 gene was upregulated in the two highest dosages as well. Meanwhile, *T. chuii* supplementation caused upregulation of SOD1 and SOD2 genes that encode for SOD except in CAF + 17 where only the SOD1 gene was upregulated. Therefore, *T. chuii* supplementation enhances the antioxidant defense mechanisms in the liver. Additionally, the CAF diet increased pro-inflammatory and inflammation-mediating genes expressions such as HMOX1, TGFβ1 and NFκB1 genes in the liver, IL-1β and TNFα genes in the mesenteric white adipose tissue (MWAT), TNFα, NRF2, HMOX1 and NFκB1 genes in the thymus, HMOX1 and NFκB1 genes in the spleen. However, they were downregulated by all dosages of *T. chuii*. Generally, these genes are involved in modulating the transcription and function of inflammatory mediators to either promote the development of inflammation [71,72]. This effect was further enhanced when the anti-inflammatory IL-10 gene that was involved in the inhibition of synthesis of pro-inflammatory cytokines synthesis [73] was upregulated in the MWAT (only in CAF + 17), thymus and spleen. On top of that, IL-1β gene in the thymus and spleen, and IFNG gene in the MWAT, thymus and spleen were downregulated. In addition, *T. chuii* supplementation upregulated the ACDC gene in the MWAT. WATs are the major source of inflammation-causing IR seen in MetS linked-obesity [74,75]. The ACDC gene is targeted for MetS because it has been shown to alleviate inflammation and diabetes and increase insulin sensitivity. ACDC gene is reported to increase the expression of IL-10 which is anti-inflammatory [76]. So, this could explain the upregulation of the IL-10 gene in MWAT, thymus and spleen following *T. chuii* supplementation. In addition, the FOXP3 gene, which is essential in regulating regulatory T cells, is involved in chronic inflammation [77]. In this study, this gene is upregulated by *T. chuii* supplementation but only in the spleen of CAF + 17 rats. Hence, *T. chuii* supplementation shows anti-inflammatory and immune system-modulating properties that alleviate MetS. A total of 17 mg/kg BW per day of *T. chuii* supplementation seems to be the best dosage in this study, but future studies should include a range of higher dosages applied in early obesity stages to evaluate the optimum dosage and its preventative effects on MetS.

#### 2.2.2. *Arthrospira platensis* (spirulina, Sp)

Lugarà et al. studied the effects of *Arthrospira platensis* (prokaryotic cyanobacterium, spirulina, Sp) supplementation on the domestic pig with Western diet (WES)–induced metabolic disturbances during gestation and lactation [60]. The 5.6 months old domestic pigs were divided into 4 groups: control diet (CTR−), control diet supplemented with 20 g/d Sp tablet (CTR+), Western diet (WES−) and Western diet supplemented with 20 g/d Sp tablet (WES+). WES recapitulates eating patterns in Westernized societies that comprise processed foods that are high in refined sugars and animal fat but low in fiber, micronutrients, and antioxidants. For this reason, it is similar to the CAF diet used to induce obesity, Mets and other metabolic alterations in animal models [65]. However, the WES diet may be more nutritionally adequate and reproducible compared to the CAF diet [78]. In this experiment, the WES indeed contained more sugar, saturated fat and calories compared to the control diet (CTR). This study employed the transcriptomic approach to reveal the expression of the genes related to the phenotypic changes caused by the WES diet and Sp supplementation. BW is one of the criteria for assessing abdominal obesity which has been identified as a risk factor for MetS [3,79]. The author has stated that the induction of metabolic disturbances through feeding of WES was only partly successful. In that context, the WES diet was not able to induce obesity, as the BW was observed to have not changed significantly among the diet groups. The author speculated that perhaps the domestic pigs used in this study were genetically less susceptible to metabolic alterations such as an increase in BW and adiposity [80]. Further, it was stated that the pigs may have become metabolically flexible in that they utilized fatty acids instead of glucose for energy, so this may have prevented adverse metabolic alterations [81]. In addition, the author noticed that the CTR diet was higher in starch compared to the WES diet which could have resulted in similar disturbances caused by the WES diet [82]. Although there was no effect on BW, the analysis of gene expression in the skeletal muscles of supplemented pigs suggests the inhibition of muscular weight gain in supplemented groups. Moreover, the supplementation did not affect the visceral adipose tissue proportion. High plasma TG is one of the risk factors involved in MetS, but it too was not affected by Sp supplementation. Additionally, the plasma TC was not affected by the supplementation. Liver weight is a crucial parameter in the assessment of diet-induced obesity which is a component of MetS and alleviation in liver weight is associated with improvement in metabolic alterations [83,84]. In this study, the liver weight was decreased by the Sp supplementation but the hepatic lipid and glycogen concentrations which are responsible for liver weight differences were similar when compared to the non-supplemented groups.

These results were not in line with the transcriptomic findings that suggest higher lipid and lower carbohydrate concentrations in the liver due to higher activation of hepatic lipid accumulation and lower activation of hepatic carbohydrate accumulation in WES+ compared to CTR- and CTR+. Hence, the Sp-mediated decrease in liver weight remains unexplained. Alanine transaminase (ALT) is one of the liver enzymes and its increase may be connected to an increase in MetS risk [85]. Additionally, an increase in ALT is often due to liver injury [86]. Surprisingly, ALT level was lower in CTR+ compared to CTR− but higher in WES+ compared to WES− animals. A similar irony was reported in the hepatic necrosis gene expression where inhibition in CTR+ but activation in WES+ was noticed. Therefore, these were denoted as a diet-dependent effect of Sp. Hence, the author concluded that Sp does not provide hepatoprotective effects but aggravates liver injury when supplemented with WES. Sp reduced the serum glucose concentration in the supplemented groups compared to the non-supplemented groups but only at the late gestation period. Furthermore, Sp reduced plasma insulin level in WES+ to similar levels observed in control diet groups which agrees with the gene expression analysis in the liver that suggests the inhibition of IR in the liver by Sp supplementation. The author concluded that Sp supplementation is partly able to alleviate or prevent the metabolic disturbance induced by the WES as it did not result in marked attenuation in several MetS parameters. The authors suggest that future studies should use animal species that are more sensitive to expressing obesity phenotypes or MetS criteria. Alternatively, the control diet could also be optimized to a healthier diet because the control diet used in this experiment was high in certain nutrients that could increase the parameters associated with metabolic disturbances, thus masking the effects of WES on the animal model. Moreover, more tissue samples could be analyzed to validate some of the alterations during gestation and lactation.

#### 2.2.3. *Diacronema lutheri*

Mayer et al. studied the effects of *Diacronema lutheri* (eukaryotic microalgae) on high-fat diet-induced MetS in rats [61]. Three week old male Wistar rats were assigned to 3 groups: a group fed with the control diet (CTRL), a group fed with a high-fat diet with 10% fructose in drinking water (HF), a group fed with a high-fat diet and 10% fructose in drinking water supplemented with 12% (w/w) of *D. lutheri* powder (HF-Dia) for 8 weeks. HF diet is commonly used to induce IR, obesity, dyslipidemia and MetS in rodent models. As its name suggests, the HF diet is enriched in fats derived from either lard, coconut or olive oil that separates the HF diet into a few types [87,88]. Due to the difference in fat source, the efficacy of each HF diet type in inducing obesity or MetS may vary [88]. The HF diet in this study contained 8 times more lipids and no dietary fibers compared to the CTRL diet. The results from this study show that HF rats had significantly higher BW compared to CTRL rats. Furthermore, the HF rats exhibited higher abdominal adipose tissue (AAT) and epidydimal adipose tissue (EAT) to BW ratio compared to CTRL rats. AAT and EAT are types of AT found within the abdominal cavity that are often related to obesity and metabolic disturbances [89,90]. Therefore, their quantification can help to evaluate abdominal obesity. AT undergoes hypertrophy causing the release of pro-inflammatory adipokines. This causes the recruitment of macrophages which also releases pro-inflammatory cytokines leading to insulin signaling impairment such as a decrease in the activation of the GLUT-4 receptor that may lead to IR [11,91,92]. These changes observed in the HF diet rats confirm that the diet has induced MetS in the rats. Supplementation of *D. lutheri* in the HF diet attenuated and prevented those detrimental effects. The AAT and EAT to BW ratios were also improved to be similar to the ratios seen in the CTRL group.

In this study, *D. lutheri* was characterized to identify its bioactive compounds. As such, the preventative effects observed by its supplementation were speculated to be due to DHA [93,94], fucoxanthin [95] and fucosterol [96] present in *D. lutheri* that exert anti-inflammatory, anti-atherogenicity and antioxidative effects. The plasma TG level was increased in HF rats but was decreased in *D. lutheri* supplementation. Conversely, the plasma HDL-C level was increased following *D. lutheri* supplementation. These findings suggest that *D. lutheri* may improve lipid metabolism and hypercholesterolemia due to the bioactivity of the bioactive compound present in *D. lutheri*. This study included atherogenic index of plasma (AIP) measurements of experimental groups. AIP is calculated according to the formula, log (TG/HDL-C). High AIP values observed in MetS, hypertension, and T2DM are due to dyslipidemia such as high plasma TG and low HDL-C. Etiologically, TG contributes to the formation of atherosclerotic plaque, while HDL-C is anti-atherosclerotic, so high AIP values increased the risk for CVD and even MetS [97]. *D. lutheri* supplementation decreased the AIP which could be due to the actions of those bioactive compounds too, suggesting the preventative potential of *D. lutheri* in developing atherosclerosis.

To elucidate the mechanism underlying the antioxidative effects of *D. lutheri*, certain plasma and adipocyte markers measurements were included in this study. Results show *D. lutheri* supplementation increased anti-inflammatory IL-10 and IL-4 levels compared to the CTRL group. This shows the anti-inflammatory capabilities of *D. lutheri* supplementation in alleviating MetS. In addition, *D. lutheri* also reduced plasma insulin, leptin, HOMAR-IR index and improved GT and IT. These findings show that *D. lutheri* supplementation could be anti-diabetic by improving insulin sensitivity. However, the glucose level was not decreased by the *D. lutheri* supplementation. In addition, the liver TG level was greatly decreased in HF-Dia compared to other diet groups while the liver TC level was reduced to be similar to the level observed in the CTRL group. These 2 liver parameters were greatly increased in HF. Therefore, *D. lutheri* may also improve NAFLD. In contrast, the ALT level was increased following the supplementation which may indicate hepatotoxicity [85]. So, future studies shall optimize the dosage to maximize benefits and minimize adverse effects.

#### 2.2.4. *Nannochloropsis oceanica*

Preez et al. investigated the effect of *Nannochloropsis oceanica* (eukaryotic microalgae) on high-carbohydrate, high-fat diet-induced MetS metabolic in rats to assess its potential as a health supplement [62]. Eight to nine week old male Wistar rats were assigned to 4 groups: a group fed with a corn starch diet (C) or high-carbohydrate, high-fat diet (H) for 16 weeks and a group fed with corn starch diet (CN) or high-carbohydrate, high-fat diet for the first 8 weeks and continued with 5% *N. oceanica* powder supplementation for the last 8 weeks (HN). H diet is used to induce MetS, obesity and IR which includes foods rich in sugar such as sucrose or fructose, and animal fat or oils. An advantage to this diet is that it is easier to standardize but may not be able to recapitulate unhealthy eating patterns that often include a variety of foods and sensory properties [98]. In this study, the H diet contained a higher amount of carbohydrates (68%, mostly fructose) and higher saturated and *trans*-fat (24% beef tallow) compared to the C diet. At the end of the 16 weeks, the H diet increased BW, plasma TG and systolic blood pressure (SBP) compared to the C diet. *N. oceanica* supplementation had no effect on BW but managed to decrease the total abdominal fat and retroperitoneal fat in HN rats compared to H rats but the visceral adiposity percentage was not attenuated by the supplementation. Although the plasma TG was elevated in the H rats, it was not reduced by supplementation. Additionally, the plasma TC was not different between any of the groups, so the supplementation certainly did not affect these parameters. Therefore, *N. oceanica* supplementation did not exhibit any preventative effects on dyslipidemia. On top of that, insulin sensitivity was reduced by the H diet, but the supplementation did not improve it. Moreover, the supplementation did not affect SBP, which was increased by the high carbohydrate, high-fat diet. Blood pressure (BP) is one of the direct criteria for diagnosing MetS, so, the results in this study reveal that *N. oceanica* supplementation did not impart any hypotensive effects.

Gut microbiota and gut health have been associated with systemic inflammation and MetS [99]. The *N. oceanica* supplementation increased the abundance of Oxyphotobacteria. Overall, the alteration in gut microbiota observed in supplemented groups was suggested to have aided in the curative effects on certain metabolic disturbances. In this study, *N. oceanica* supplementation decreased the size of fat vacuoles in the liver which suggests an improvement in lipid metabolism [94]. Additionally, *N. oceanica* was characterized for its nutrient composition and *N. oceanica* contains high amounts of PUFAs such as EPA, vitamins, and carotenoids. The author stated that EPA and antioxidants in *N. oceanica* could be responsible for some of the alleviation observed in the MetS models of previous studies [63,94]. Hence, *N. oceanica* was concluded as a potential prebiotic, source of essential nutrients and anti-inflammatory with the potential to alleviate NAFLD. Future studies can employ methods to disrupt the cell and increase the bioavailability of bioactive compounds in the microalgae that could result in better attenuation of metabolic disturbances [100]. Moreover, the effect of *N. oceanica* on NAFLD and NASH can be studied too since a decrease in fat vacuole sizes was observed in the supplemented groups. Furthermore, gene expression studies can be conducted to explain the mechanisms underlying the decrease in fat mass.

#### 2.2.5. *Tisochrysis lutea*

Mayer et al. studied the effect of *Tisochrysis lutea* (eukaryotic microalgae) on metabolic disturbances associated with MetS and obesity using HF diet-induced MetS rat models. Three week old male Wistar rats were assigned to 3 groups: rats fed with a standard diet (CTRL), rats fed with an HF diet with 10% fructose in drinking water (HF) and rats fed with an HF diet with 12% *T. lutea* powder (HF-Tiso) for 8 weeks each [6]. HF diet caused the rats to have the highest BW, AAT and EAT weight to BW ratios, plasma glucose, insulin, leptin, LDL-C, HOMA-IR index, AIP value and the lowest HDL-C level compared to other groups. *T. lutea* supplementation decreased the BW in HF-Tiso rats. Other than that, the supplementation decreased the AAT and EAT weight to BW ratio. The BW and adipose tissue weight attenuation by *T. lutea* is associated with its bioactivity of DHA. DHA is said to activate PPARα/γ, thus improving lipid metabolism [93]. Furthermore, fucoxanthin present in *T. lutea* may have alleviated the BW and adipose tissue weight as well by increasing the utilization of fat for energy [101]. In parallel, *T. lutea* supplementation decreased plasma TG and LDL-C levels and increased HDL-C level. It has become obvious that high plasma TG and low HDL/LDL ratio are prominent risk factors for developing cardiovascular diseases. Additionally, *T. lutea* supplementation decreased AIP, further corroborating these plasma lipid findings. These cardioprotective effects and alleviation in dyslipidemia were some of the therapeutic potentials of n-3 LC-PUFAs such as DHA [94], phytosterols [102], and soluble fibers [103] found in *T. lutea*. In addition, *T. lutea* supplementation caused a decrease in plasma glucose and insulin levels which suggests the supplementation may protect against IR which was confirmed by the improvement in the HOMA-IR index. These effects were achieved, attributed to DHA, fucoxanthin, and their role in improving hyperglycemia [63,101]. Systemic inflammation, a condition common in MetS was alleviated by *T. lutea* supplementation. The supplementation also lowered levels of pro-inflammatory plasma TNF-α and higher levels of anti-inflammatory IL-10 in adipose tissue. LPS, an endotoxin from bacteria in the gut microbiome leaks into the blood circulation and its chronic exposure may induce systemic inflammation leading to MetS. This condition is known as metabolic endotoxemia [99]. HF-Tiso group rats show decreased levels of serum LPS that indicate a reduction in the degree of systemic inflammation by *T. lutea* supplementation. The author speculated that fucoxanthin [101] and DHA [94] exert anti-inflammatory effects by pro-inflammatory pathway inhibition or decreasing the synthesis of pro-inflammatory cytokines while increasing the synthesis of anti-inflammatory cytokines. The author also stated that other antioxidants could also enhance this effect. A high leptin level is an indicator of a pro-inflammatory state and is correlated to increased adiposity [104]. Plasma leptin level was decreased in HF-Tiso, showing an alleviation in systemic inflammation due to the bioactivity of DHA and fucoxanthin found in *T. lutea*. NAFLD, characterized by hepatic steatosis has been identified as one of the manifestations of MetS [105]. In this study, the supplementation improved certain markers of NAFLD. The liver TG and TC were also decreased by *T. lutea* supplementation. This shows the hepatoprotective potential of *T. lutea* which is attributed to DHA and EPA [93,94]. The author explained that DHA improves lipid metabolism, thus, reduces lipid accumulation in the liver [93,94]. In parallel, the presence of fucoxanthin in *T. lutea* could also exhibit such bioactivity [95].

#### 2.2.6. DHA-Rich Microalgae Mixture

Elzinga et al. studied the effects of DHA-rich microalgae supplementation on metabolic and inflammatory responses using horses with Equine Mets (EMS) as the model to represent MetS [63]. The EMS horses were assigned to 2 groups: control horses and treated horses that were supplemented with DHA-rich microalgae for 46 days. Tests to measure parameters related to MetS were performed on day 0 and day 46. In that context, the BW and cresty neck scores (including fat deposition levels) in the horses show no difference between treated and control horses. However, the results also show that the plasma TG levels decreased over time in the treated horses compared to control horses. Plasma DHA levels of 46-days-treated horses were found to be elevated markedly. This is because the microalgae contain DHA, and it has been suggested to have anti-inflammatory effects that can improve insulin sensitivity [47]. Nevertheless, the supplementation had no effects on glucose tolerance. Therefore, this finding does not confirm the curative effects of DHA-rich microalgae on IR in EMS. However, the author concludes that DHA-rich microalgae may improve insulin sensitivity. Through gene expression studies, it was found that the pro-inflammatory TNF-α cytokine gene expression was decreased in treated horses at day 0 and was even lower at day 46 compared to control horses. The author suggested that DHA in the microalgae is responsible for this anti-inflammatory effect [93]. In this work, it was also reported that the DHA content remained significantly elevated after the experimental periods because DHA could be stored in adipose tissues and be released over a long period of time, especially in MetS models that typically have high adiposity. In the future, more parameters that are associated with MetS shall be measured such as plasma cholesterol, visceral adiposity, and BP.

#### 2.2.7. *Phaeodactylum tricornutum*

Mayer et al. studied the potential of *Phaeodactylum tricornutum* (eukaryotic microalgae) as a supplement to alleviate disturbances associated with MetS in rats with HF diet-induced MetS for 8 weeks [10]. Three week old male Wistar rats were assigned to 3 groups: a group fed with a standard diet as the control (CTRL), a group fed with HF diet including 10% fructose in drinking water (HF), and a group fed the same HF diet but supplemented with 12% *P. tricornutum* powder (HF-Phaeo). The BW and adipose tissue weight of HF group rats were the highest compared to other groups, which confirms that the HF diet induces these signs of MetS. The BW of HF-Phaeo rats decreased to a similar BW of CTRL group rats. Additionally, AAT and EAT weight to BW ratio was also lower in HF-Phaeo. Despite the rats having similar energy intake in HF and HF-Phaeo groups, the BW and adipose tissue weight were still improved by the supplementation, emphasizing the preventative effects of *P. tricronutum* supplementation on BW and adiposity elevation caused by the HF diet. To explain these findings, quite an extensive FA profiling of lipids in plasma, RBC and liver was conducted to connect the changes observed in the FA composition to the physiological changes exerted by *P. tricornutum* supplementation. It was found that *P. tricornutum* has abundant levels of EPA, thus, its supplementation increased the n-3 LC-PUFA levels in plasma, RBC, and liver lipids in HF-Phaeo. The increase in tissue n-3 LC-PUFA, especially EPA, is linked to improvement in BW and fat mass. EPA could exert this effect due to a reduction in hypertrophy and hyperplasia of adipocytes. This is also linked to a decrease in BW and fat mass observed in this study [93]. Aside from EPA, fucoxanthin is also found in *P. tricorntunum* which could aid in BW and fat mass reduction attributed to improving energy expenditure, β-oxidation and adipogenesis [95]. Additionally, the dietary fibers of *P. tricornutum* may have added to those preventative effects [103].

Abnormal FA composition is a trending feature of MetS [106] and increased MUFA levels were observed in rats fed a high-sugar diet [107]. The degree of FA unsaturation is related to lipotoxicity in hepatic steatosis which is closely associated with MetS [107,108]. In this study, *P. tricorntunum* supplementation may improve hepatic FA composition indicated by the decrease of MUFA levels in liver phospholipids due to a reduction in ∆9-Desaturase levels in liver lipids of HF-Phaeo. ∆9-Desaturase is the rate-limiting enzyme in the synthesis of MUFA [109]. Moreover, the supplementation caused a reduction in the liver weight to BW ratio. These results show that *P. tricornutum* supplementation may protect the liver against the steatosis that characterizes NAFLD. On top of that, the MUFA levels were also reduced in plasma. The plasma and hepatic TC and TG levels were significantly reduced in HF-Phaeo which shows that *P. tricorntunum* supplementation may correct dyslipidemia in MetS. Moreover, *P. tricorntunum* supplementation increased plasma HDL levels. EPA regulates lipid metabolism by inhibiting lipogenesis and TG secretion [94]. This underlies the attenuation of dyslipidemia in MetS by *P. tricornutum* supplementation. However, synergistic effects from fiber, phytosterols and fucoxanthin may also play a part. Due to that, *P. tricorntunum* supplementation lowered the AIP score which indicates a lower risk of developing atherosclerosis and abdominal obesity. Additionally, the enrichment of RBC phospholipid with n-3 LC-PUFA is associated with the decreased AIP value. *P. tricornutum* alleviates inflammation by decreasing plasma pro-inflammatory TNF-α and IL-6 levels. Meanwhile, plasma and adipose anti-inflammatory IL-4 and IL-10 levels were increased by supplementation. High leptin level observed in the HF group indicates pro-inflammatory condition and is linked to body fat accumulation. By contrast, plasma leptin, insulin levels and HOMA-IR index were reduced following *P. tricorntunum* supplementation, but plasma glucose levels were not affected by the supplementation. This shows *P. tricornutum* improves insulin sensitivity.

#### 2.2.8. *Coccomyxa gloeobotrydiformis*

Zheng et al. investigated the therapeutic effects of *Coccomyxa gloeobotrydiformis* (CGD, eukaryotic microalgae) supplementation on MetS by using a diet-induced MetS model in rats [64]. The rats were assigned to 6 groups: a group fed with standard chow diet (control), a group fed with high-energy diet for 16 weeks without CDG supplementation (NC), a group fed with high-energy diet for 16 weeks without CDG supplementation (MS), a group fed with high-energy diet for 16 weeks without cardiovascular complications and CDG supplementation (MS + CGD), a group fed with high-energy diet for 16 weeks without CDG supplementation (MS + CVD) and a group fed with a high-energy diet for 16 weeks with cardiovascular complications and CDG supplementation (MS + CVD + CGD). In this study, the high-energy diet consisted of 52.5% standard laboratory chow topped up with 20% lard, 10% sugar and 10% dry milk solution which make it to be more energy-dense compared to the control diet. This diet has been used to induce MetS and obesity because excess energy intake leads to metabolic alterations [110,111]. Results show that MS + CGD caused a reduction in BW, abdominal circumference (AC), serum glucose and SBP compared to MS. In terms of serum lipid levels, CGD supplementation decreased TG and LDL-C levels while the HLD-C level was increased in MS + CGD. The improvement in these biochemical parameters evidenced the curative effects of CDG in ameliorating the disturbances associated with MetS.

Mitochondrial dysfunction is one of the mechanisms involved in the pathogenesis of MetS in which AMPK and PGC-1α activities are decreased. Through the AMPK/PGC-1α pathway, mitochondrial and energy metabolism is regulated [112]. In the heart, adipose tissue, and skeletal muscle of MS + CGD rats, AMPK and PGC-1α gene expressions were increased compared to the MS rats that underlie the therapeutic effects of CGD supplementation in MS + CGD rats. Impairment in ATP production is related to heart failure, T2DM and MetS. Mitochondrial respiratory chain (MRC) enzymes attempt to produce ATP through oxidative phosphorylation to regulate energy metabolism [113]. It was found that CGD supplementation increased expressions of MRC coenzymes such as ATPase 6, cytochrome b and SDHA in the liver, heart, and skeletal muscle of MS + CGD rats. In contrast with the other MRC coenzymes, UCP2 negatively affects glucose and lipid metabolism by reducing ATP synthesis and insulin synthesis [114]. Its expression was decreased by CGD supplementation. Therefore, CGD supplementation improves mitochondrial function leading to the alleviation of MetS by improving energy metabolism. In this study, the specific effect of CGD supplementation on CVD caused by MetS was also investigated. Compared to the control, the MS + CVD + CGD group had decreased myocardial tissue expressions of pro-inflammatory TNF-α, and malondialdehyde (MDA) which is a marker of oxidative stress associated with metabolic alterations and inflammation [115]. In addition, SOD, which functions as a free radical scavenger [115], had increased expression in MS + CVD + CGD compared to the control. Therefore, CGD supplementation acts as an anti-inflammatory and antioxidant agent in alleviating CVD in MetS. The authors also postulated that dysregulated apoptosis caused by oxidative stress in cardiomyocytes may also lead to CVD [115]. In MS + CVD + CGD, the expression of the anti-apoptotic Bcl-2 gene was increased but the pro-apoptotic Bax gene and cleaved caspase-3 were decreased when compared to the control group. In addition, CGD supplementation also caused an increase in TMOD1 expression, which is a protein that regulates cardiomyocyte physiology in MS + CVD + CGD rats compared to MS + CVD rats. Furthermore, cardiac function test parameters such as left ventricular systolic and end diastolic pressure, and left ventricular systolic pressure maximum increase rate and diastolic pressure maximum decrease rate were increased in MS + CVD + CGD compared to MS + CVD. This shows that CGD supplementation in MetS with cardiovascular complications improves cardiac function. According to the authors, the therapeutic effects of CGD supplementation reported in this study are perhaps due to its anti-inflammatory, antioxidative and anti-apoptosis properties. Furthermore, CGD supplementation can improve energy metabolism and regulate mitochondrial function.

## 3. Limitation

Currently, there are a few limitations to applying microalgae as a health supplement. Firstly, the mechanisms of action underlying the therapeutic effects of microalgae algae on MetS have been the subject of only some of the studies. Moreover, only a small number of microalgae species have been studied for their effects on MetS and there has only been a single paper studying each microalgae species. Most of the studies focused on their preventative effects so there is little supporting data on their curative effects if they are to be compared with existing drugs to treat MetS. Additionally, the parameters measured, or the metabolic disturbances observed, were not the same among the studies. For example, most of the studies did not measure BP, although hypertension is one of the risk factors of MetS. So, the effects of some of the microalgae on certain components of MetS are largely unknown. On top of that, some of the microalgae compositions in these studies were not consistent with previous studies which are possibly due to the differences in culture conditions. Therefore, this affects the concentration of microalgae biomass and bioactive compounds. Moreover, most of the studies only applied a single set of microalga dosages, but the dosage is based on a previous work that studied different microalgae species. Only three out of the nine studies evaluated the digestibility or bioavailability of the respective microalgae extracts, which is an important factor considering that intact cell wall of microalgae may reduce the bioavailability and full effect of bioactive compounds. Additionally, there is a lack of studies investigating the hepatotoxicity of microalgae when applied as a health supplement. So, the therapeutic index of microalgae in these studies may not be at the optimum level yet. Furthermore, no human studies have been conducted that would have deemed the findings to be more relevant for humans.

## 4. Future Perspective

**Figure 1:**
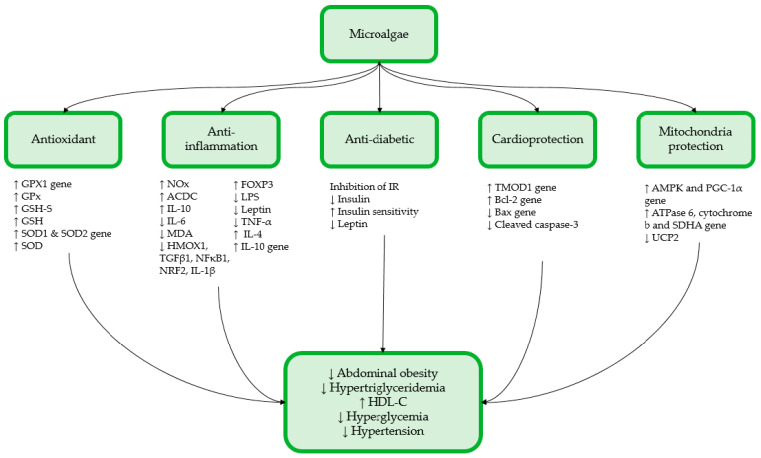
Summary of the potential therapeutic effects of microalgae on the components of MetS.

Future studies shall focus on elucidating the mechanism of actions underlying the therapeutic effects of microalgae on alleviating metabolic disturbances associated with MetS by the molecular targets and identifying the activated genes or pathways and the proteins expressed in the presence of a particular microalgae bioactive compound. In this review, microalgae supplementation has been shown to exert curative and/or preventative effects on several components of MetS such as abdominal obesity, dyslipidemia and improves insulin sensitivity (Figure 1). These improvements were attributed to the bioactive compounds found in microalgae that are associated with their antioxidant and anti-inflammatory activities. Although the bioactive compound types and levels in every microalga species are not the same, each microalgae species comprises a unique composition of bioactive compounds that alleviate a certain metabolic disturbance. To maintain a consistent and optimum composition of the desired bioactive compound and biomass within a microalgae species, culture conditions need to be ideal and consistent. Therefore, further studies in this area are required to establish an optimum culturing protocol so microalgae can be successfully transformed into marketable and efficacious health supplements. Moreover, the effects of microalgae supplementation on all components of MetS or some of the components missed out in these studies can be performed to fully assess the potential of microalgae as a multi-target supplement. As expected, most of the microalgae dosages applied for alleviating MetS did not exert any adverse side effects. Nevertheless, existing pharmacological drugs exhibit better efficacy than some of the microalgae supplementations in alleviating metabolic disturbances. Therefore, the current findings suggest that microalgae supplementation would be better at preventing the development of metabolic disturbances associated with MetS, but its curative potential is still significant. So, future studies can apply larger dosages of microalgae supplementations together with toxicity studies to optimize its efficacy. Hence, combined with the increasing prevalence of MetS and the growing demand for healthy foods, microalgae could be a prominent health supplement in the coming future. However, future research shall translate current findings into human trials to obtain a more relevant evaluation on the efficacy of microalgae supplementation in alleviating MetS.

## Figures and Tables

**Table 1:** The effects of microalgae supplementation on MetS-related cell model.

Experiment	Cell Model	Microalgae and Doses	Experimental Groups	Effects	Mechanisms	Reference Number
Dose-response analysis	COS-1 cells	0.1% *Chaetoceros karianus*-derived (7E)-9-OHE or (10E)-9-OHE for 18 h	(1) Positive controls: rosiglitazone or pirinixic acid (2) (7E)-9-OHE or (10E)-9-OHE (3) Negative control: palmitic acid	(7E)-9-OHE or (10E)-9-OHE: Exhibits PPARα/γ agonist activity	-	[38]
Endogenous PPAR target genes activation analysis	Huh7 cells SGBS pre-adipocyte cells	25 or 50 µM (7E)-9-OHE or (10E)-9-OHE for 24 h 2 µM rosiglitazone for 24 h	(1) DMSO (negative controls) (2) 25 µM (7E)-9-OHE or (10E)-9-OHE (3) 50 µM (7E)-9-OHE or (10E)-9-OHE (4) pirinxic acid (positive control)	25 or 50 µM of (7E)-9-OHE or (10E)-9-OHE: Fatty acid catabolism is activated in Huh-7 and SGBS cells	Huh-7: (7E)-9-OHE: ↑ ACSL3 gene expression (10E)-9-OHE: ↑ PLIN1 gene expression (7E)-9-OHE or (10E)-9-OHE: ↑ CPT1A and ANGPTL4 gene expressions SGBS: (10E)-9-OHE: ↑ ANGPTL4 gene expression (7E)-9-OHE or (10E)-9-OHE: ↑ CPT1A gene expression	
Adipocyte differentiation analysis	SGBS pre-adipocyte cells	25 µM of (7E)-9- OHE or (10E)-9-OHE for 12 days	(1) (7E)-9-OHE or (10E)-9-OHE (2) rosiglitazone (positive control)	Improvement in the regulation of fatty acid metabolism, transport, storage, adipokine signaling and browning	↑ PPARG, CEBPA, CEBPB, PLIN1, FABP4, CD36, SCD1 and UCP1 expressions	
Adipocyte transcriptomics	SGBS pre-adipocyte cells	25 µM of (7E)-9-OHE or (10E)-9-OHE for 8 days	(1) (7E)-9-OHE or (10E)-9-OHE (2) rosiglitazone (positive control)	↓ Inflammatory cytokines ↑ Insulin-sensitive adipokines	↓ IL-6, TNFα, CXCL1, CXCL5 and IL-1B gene expressions ↑ Leptin and insulin sensitizing ADIPOQ genes expressions	

ACSL3: acyl-coA synthetase long chain family member 3, ADIPOQ: adipokine adiponectin, CD36: cluster of differentiation 36, CEBPA: CCAAT enhancer-binding protein alpha, CEBPB: CCAAT enhancer-binding protein beta, CPT1A: carnitine palmitoyltransferase 1A, CXCL1: CXC motif chemokine ligand 1, CXCL5: CXC motif chemokine ligand 5, FABP4: fatty acid binding protein 4, IL-1B: Interleukin-1 beta, IL-6: interleukin-6, OHE: oxohexadecenoic acid, PLIN1: perilipin 1, PPAR: peroxisome proliferator-activated receptor, PPARG: peroxisome proliferator-activated receptor gamma, SCD1: Stearoyl-CoA desaturase 1, SGBS: Simpson–Golabi–Behmel syndrome, TNFα: tumor necrosis factor α, UCP1: uncoupling protein 1.

**Table 2:** The effects of microalgae supplementation on MetS-induced animal models.

Animal Model	Microalgae and Doses	Experimental Design	Effects on MetS	Mechanisms	Reference Number
Male Sprague Dawley rat (7 weeks old)	*Tetraselmis chuii* powder (0.17, 1.7, 17 mg/kg BW/d) 8 weeks	STD-C: Standard diet-control CAF-C: Cafeteria diet- control CAF + 0.17: CAF + 0.17 mg/kg BW/day of *T. chuii* powder CAF + 1.7: CAF + 1.7 mg/kg BW/day of *T. chuii* powder CAF + 17: CAF + 17 mg/kg BW/day of *T. chuii* powder	CAF + 0.17: ↓ plasma LDL/VLDL-C CAF + 17: ↓ Plasma glucose CAF + 0.17, CAF + 1.7 and CAF + 17: No effects on BW, adiposity index, TG, HOMA-IR index, and HDL-C.	CAF + 0.17: ↑ plasma NOx ↑ GPx activity in liver CAF + 0.17 and CAF + 1.7: ↑ SOD 1 and SOD2 gene expression in liverCAF + 1.7 and CAF + 17: ↑ GPX1 gene expression in liver ↑ GCLm gene expression in liver CAF + 17: ↓ oxLDL levels in plasma ↑ IL-10 levels in plasma ↑ GSH level in liver ↑ SOD1 gene expression in liver ↑ IL-10 gene expression in MWAT ↑ FOXP3 gene expression in spleen CAF + 0.17, CAF + 1.7 and CAF + 17: ↑ GR and GSH-S gene expressions in liver ↑ SOD1 gene expression in liver ↓HMOX1, TGFβ1 and NFκB1 gene expressions in liver ↓ IL-1β, TNFα and IFNG gene expressions in MWAT ↓ IL-1β agene in thymus and spleen ↓ IFNG gene expressions in MWAT, thymus and spleen ↑ ACDC gene expression in MWAT ↓ TNFα, NRF2, HMOX1, NFκB1, IL-1β and IFNG gene expressions in thymus ↑ IL-10 gene expression in thymus and spleen	[59]
Female Sus scrofa pigs(5.6 ± 0.8 months old)	*Arthrospira platensis* (spirulina, Sp) (20 g/d) tablet 25 weeks	CTR−: Control diet CTR+: Control diet + Sp tablet WES−: Western diet WES+: Western diet + Sp tablet	CTR+ and WES+: No effects on BW No effects on visceral adipose tissue proportion No effects on plasma TG No effects on plasma TC ↓ Serum glucose (at late gestation)	CTR+: ↓ ALT levels ↓ Hepatic necrosis gene expression WES+: ↑ Hepatic lipid accumulation gene expression ↓ Hepatic carbohydrate accumulation gene expression ↑ ALT levels ↑ Hepatic necrosis gene expression CTR+ and WES+: ↓ Plasma insulin levels (at slaughter) ↓ Muscular weight gain gene expression ↓ Liver weight ↓ IR gene expression in liver	[60]
Male Wistar rat (3 weeks old)	*Diacronema lutheri* powder (12%) 8 weeks	CTRL: Control diet HF: High fat diet HF-Dia: High fat + *D. lutheri* powder	HF-Dia: ↓ BW ↓ AAT and EAT weight/BW ratio ↓ Plasma TG levels ↑ HDL levels ↓ HOMA-IR index Improvement in GT & IT No effects on plasma glucose levels	HF-Dia: ↓ Plasma insulin levels ↓ AIP ↑ Plasma IL-4 levels ↑ Adipose IL-10 levels ↓ Leptin ↓ TG in liver ↓ TC in liver ↑ ALT ratio	[61]
Male Wistar rat (8–9 weeks old)	*Nannochloropsis oceanica* powder (5%) 8 weeks	C: Corn starch diet for 16 weeks H: High-carbohydrate, high-fat diet for 16 weeks CN: Corn starch diet for the first 8 weeks + 5% *N. oceanica* powder for the last 8 weeksHN: High-carbohydrate, high-fat diet for first the 8 weeks + 5% *N. oceanica* powder for the last 8 weeks	HN: ↓ Total abdominal fat and retroperitoneal fat No effects on BW CN and HN groups: No effects on visceral adiposity % No effects on plasma TG No effects on plasma TCNo improvement in GT & IT No effects on systolic BP	HN: ↓ Hepatic fat vacuole size CN and HN groups: ↑ Abundance of Oxyphotobacteria	[62]
Male Wistar rat (3 weeks old)	*Tisochrysis lutea* powder (12%) 8 weeks	CTRL: Standard diet HF: 260 High fat diet with 10% fructose in drinking water HF-Tiso: HF diet + *T. lutea* powder	HF-Tiso: ↓ BW ↓ AAT and EAT weight/ BW ratio ↓ Plasma TG ↑ Plasma HDL-C ↓ Plasma LDL-C ↓ Plasma glucose ↓ HOMA-IR index	HF-Tiso: ↓ Plasma insulin ↓ Plasma TNF-α ↑ Adipose tissue anti-inflammatory IL-10 ↓ AIP ↓ Serum LPS ↓ Leptin ↓ TG in liver ↓ TC in liver	[6]
Mixed sex and breed of horse (treatment group is 13.2 ± 4.4 years old and control group is 11.5 ± 2.6 years old)	DHA-rich microalgae (110 g/horse/d) 46 days	Control horses Treated horses	Treated horses: No effects on BW No effect on cresty neck score ↓ Plasma TG No effects on glucose tolerance	↑ Plasma DHA ↓ TNF-α MFI	[63]
Male Wistar rat (3 weeks old)	*Phaeodactylum tricornutum* powder (12%) 8 weeks	CTRL: Control group fed with standard diet HF: 260 High fat diet with 10% fructose in drinking water HF-Phaeo: HF diet + *P. tricornutum* powder	HF-Phaeo: ↓ BW ↓ AAT and EAT weight/ BW ratio ↓ Plasma TC ↓ Plasma TG ↑ Serum HDL-C ↓ HOMA-IR index No effects on plasma glucose	HF-Phaeo: ↓ Plasma insulin ↑ n-3 LC-PUFA levels in plasma, RBC, and liver lipids ↑ ∆9-Desaturase level in liver lipids ↓ Liver weight/ BW ratio ↓ MUFA levels in plasma lipid and liver phospholipids ↓ TG in liver ↓ TC in liver ↓ AIP ↓ Plasma TNF-α and IL-6 ↑ Plasma IL-4 and IL-10 ↓ Plasma leptin	[10]
Sprague Dawley rat(8 weeks old)	*Coccomyxa gloeobotrydiformis* (CGD) (100 mg/kg BW/d) 12 weeks	Control: Standard chow diet NC: High-energy diet without MetS MS: High-energy diet with MetS MS+CGD: High-energy diet with MetS + CDG MS + CVD: High-energy diet with MetS and CVD MS + CVD + CGD: High-energy diet with MetS and CVD + CGD	MS + CGD: ↓ BW ↓ AC ↓ Serum glucose level ↓ SBP ↓ Serum TG and LDL-C levels ↑ Serum HDL-C levels MS + CVD + CGD: ↑ Left ventricular systolic and end diastolic pressure, and left ventricular systolic pressure maximum increase rate and diastolic pressure maximum decrease rate	MS + CGD: ↑ AMPK and PGC-1α gene expressions in heart, adipose and skeletal muscle tissues ↑ MRC coenzymes (ATPase 6, cytochrome b and SDHA) gene expressions in the liver, heart and skeletal muscle ↓ UCP2 gene expression MS + CVD + CGD: ↓ Pro-inflammatory TNF-α and MDA gene expressions in myocardial tissue ↑ SOD gene expression in myocardial tissue ↑ Bcl-2 gene expression ↓ Bax gene and cleaved caspase-3 gene expressions were decreased ↑ TMOD1 gene expression	[64]

AAT: abdominal adipose tissue, AC: abdominal circumference, ACDC: adiponectin, AIP: atherogenic index of plasma, ALT: alanine transaminase, AMPK: adenosine monophosphate-activated protein kinase, AST: aspartate transaminase, ATPase 6: adenosine triphosphatase 6, Bcl-2: B-cell lymphoma 2, BW: body weight, EAT: epididymal adipose tissue, FOXP3: forkhead box P3, GCLm: glutamate-cysteine ligase modifier subunit, GHS-S: GSH synthetase, GPx: glutathione peroxidase, GPx1: glutathione peroxidase 1, GR: glutathione reductase, GSH: glutathione, GT: glucose tolerance, HDL-C: high-density lipoprotein cholesterol, HMOX1: Heme oxygenase 1, HOMAR-IR: Homeostatic Model Assessment of Insulin Resistance, IFNG: interferon gamma, IL-10: interleukin-10, IL-1β: interleukin 1 beta, IL-4: interleukin-4, IL-6: interleukin-6, IR: insulin resistance, IT: insulin tolerance, LDL-C: low-density lipoprotein cholesterol, LPS: lipopolysaccharides, MDA: malondialdehyde, MFI: mean fluorescence intensity, MRC: mitochondrial respiratory chain, MUFA: monounsaturated fatty acids, MWAT: mesenteric white adipose tissue, n-3 LC-PUFA: n-3 polyunsaturated fatty acids, NEFAs: non-esterified fatty acids, NFκB1: nuclear factor kappa B subunit 1, NOx: nitric oxide metabolites, NRF2: nuclear factor erythroid 2-related factor 2, oxLDL: oxidized low-density lipoprotein, PGC-1α: peroxisome proliferator-activated receptor gamma coactivator 1-alpha, RBC: red blood cell, SBP: systolic blood pressure, SDHA: succinate dehydrogenase complex, subunit-A, SOD: superoxide dismutase, SOD1: superoxide dismutase 1, SOD2: superoxide dismutase 2, TC: total cholesterol, TG: triglyceride, TGFβ1: transforming growth factor beta-1, TMOD1: Tropomodulin 1, TNFα: tumor necrosis factor alpha, UCP2: uncoupling protein 2, VLDL-C: very low-density lipoprotein cholesterol.

## Data Availability

Not applicable.
